# Synthesis of Nanocomposites and Catalysis Applications

**DOI:** 10.3390/nano12050731

**Published:** 2022-02-22

**Authors:** Evgeny Gerasimov

**Affiliations:** Boreskov Institute of Catalysis SB RAS, 630090 Novosibirsk, Russia; gerasimov@catalysis.ru

The term catalysis was introduced in the mid-19th century by the Swedish scientist Jöns Jakob Berzelius, ushering in the era of accelerated chemical reactions. Around the same time, the English scientist Michael Faraday synthesized colloidal gold by reducing gold chloride with phosphorus, which can be considered as the synthesis of a nanomaterial. The search for substances that accelerate a particular reaction and the synthesis of nanomaterials have since developed rapidly, both in separate fields of science and in joint applications. Today, the invention of a catalyst is hard to imagine without preliminary chemical engineering, selection of a combination of initial reagents, and the modification of their properties and morphological characteristics. As mentioned in the opening sentences of this Special Issue: “Catalytic technologies are required in various industries: in the chemical and food industries, energy, wood processing, and pharmaceuticals. Catalysts are involved in 70–80% of all chemical processes. The global catalyst market size was estimated at USD 25.0 billion in 2018 and continues to grow”.

In many respects, the rapid development of nanocomposites in catalysis is due to their wide applicability, the possibility of varying their compositions and properties, and their great lability of synthesis methods. The new challenges that the world community poses to scientists are largely dictated by the development of the fields of materials science and catalysis. In this Special Issue, ***Synthesis of Nanocomposites and Catalysis Application***, we can distinguish several units corresponding to the current trends in catalysis.

We could not but have included one of the most important issues in green chemistry, i.e., the production and transport of hydrogen, in the first unit of the Special Issue. According to many scientists, this is the foundation of future energy generation. In this Special Issue, hydrogen is proposed to be obtained by dry methane reforming on Ni/Ce-Zr-O systems [1], from the hydrolysis of dimethylamine-borane (DMAB) by ultrafine Pd nanoparticles supported on soft nitriding porous carbon [2], by photocatalytic decomposition on solid solutions Cd_1−x_Mn_x_S and CdS-β-Mn_3_O_4_-MnOOH under visible light conditions [3] or with tungsten carbide (WC) in hydrogen evolution reactions [4]. One of the promising approaches to hydrogen storage and transport is the use of liquid organic hydrogen carriers, methylcyclohexane and Ni-based catalyst synthesized via the heterophase sol–gel technique, which were used for these purposes in [5].

Another important part of the Special Issue dwells upon the problem of the processing or disposal of by-products from fuel combustion, natural resource extraction, and toxic production. These emissions are very dangerous for the environment and human health. In [6], the use of catalysts based on Mn-Ce oxides is proposed as a CO neutralization catalyst. In [7], CuFeAl nanocomposite catalysts for coal combustion in a fluidized bed are used, and optimal compositions of mixtures and catalysts are considered. In the case of the utilization of methane formed during the extraction of natural resources, the process of the deactivation of catalysts occurring under the conditions of a deep oxidation reaction based on La-Ca-Mn-Co perovskites was considered in [8] by the in situ XRD method. In [9], a g-C_3_N_4_/ZnO nanocomposite was used for the degradation of 5-fluoruracil cytostatic drug under UV–LED irradiation.

Traditionally, catalysts based on nanocomposites are used in many of the synthesis processes of new and important materials for industry. The study of reaction mechanisms and possible by-products is important in obtaining new processes and products. The oxidative N-formylation of secondary amines catalyzed by reusable bimetallic AuPd-Fe_3_O_4_ nanoparticles was studied in [10]. In [11], the coupling hydrogenation of guaiacol with in situ hydrogen production by glycerol aqueous reforming over Ni/Al_2_O_3_ and Ni-X/Al_2_O_3_ (X = Cu, Mo, P) catalysts was performed.

In conclusion, I would like to thank all of the authors who contributed to this Special Issue, as well as the members of the ***Nanomaterials*** editorial board for their joint work.
